# The intention of Dutch general practitioners to offer vaccination against pneumococcal disease, herpes zoster and pertussis to people aged 60 years and older

**DOI:** 10.1186/s12877-017-0511-7

**Published:** 2017-06-07

**Authors:** Birthe A. Lehmann, Renske Eilers, Liesbeth Mollema, José Ferreira, Hester E. de Melker

**Affiliations:** 1 Center for Infectious Disease Control, National Institute for Public Health and the Environment (RIVM), Bilthoven, The Netherlands; 2 Department of Epidemiology, University of Groningen, University Medical Center Groningen, Groningen, The Netherlands

**Keywords:** General practitioners, Older people, Vaccination, Influenza, Pneumonia, Herpes zoster, Pertussis, Social cognitive predictors

## Abstract

**Background:**

Increasing life expectancy results in a larger proportion of older people susceptible to vaccine preventable diseases (VPDs). In the Netherlands, influenza vaccination is routinely offered to people aged 60 years and older. Vaccination against pneumococcal disease, herpes zoster and pertussis is rarely used. These vaccines will be evaluated by the Dutch Health Council and might be routinely offered to older people in the near future. Possible expansion of the program depends partly on the willingness of general practitioners (GPs) to endorse additional vaccinations. In this study, we assessed predictors of GPs’ attitude and intention to vaccinate people aged 60 years and older.

**Methods:**

GPs (*N* = 12.194) were invited to fill in an online questionnaire consisting of questions about social cognitive factors that can influence the willingness of GPs to vaccinate people aged 60 years and older, including underlying beliefs, practical considerations of adding more vaccines to the national program, demographics, and GPs’ patient population characteristics. The questionnaire was filled in by 732 GPs.

**Results:**

GPs were positive both about vaccination as a preventive tool and the influenza vaccination program, but somewhat less positive about expanding the current program. Prediction analysis showed that the intention of GPs to offer additional vaccination was predicted by their attitude towards offering additional vaccination, towards vaccination as a preventive tool, towards offering vaccination during an outbreak and on GPs opinion regarding suitability to offer additional vaccination (R^2^ = 0.60). The attitude of GPs towards offering additional vaccination was predicted by the perceived severity of herpes zoster and pneumonia, as well as the perceived incidence of herpes zoster. Severity of diseases was ranked as important argument to recommend vaccination, followed by effectiveness and health benefits of vaccines.

**Conclusion:**

Providing GPs with evidence-based information about the severity and prevalence of diseases, and effectiveness and health benefits of the vaccines, together with an active role of GPs in informing older people about vaccines, could modify the intention towards additional vaccination of people 60 years and older.

**Electronic supplementary material:**

The online version of this article (doi:10.1186/s12877-017-0511-7) contains supplementary material, which is available to authorized users.

## Background

Life expectancy has increased around the world, resulting in a larger proportion of older people in the population. It is estimated that by 2060, 28.4% of the population in the EU will consist of people older than 65 years of age, which would be an increase of almost 10% since 2014 [1]. Age-dependent deterioration of the immune system, called immunosenescence, together with general frailty and possible co-morbidity make older people particularly susceptible to infectious diseases [2, 3]. Infectious diseases play an important role in the disease burden of older people and an increased social engagement among community-dwelling older adults might additionally increase the risks of transmission [4–6]. Moreover, immunity – vaccine and naturally acquired – can wane over time which makes reactivation of certain latent viruses possible [7]. Successful (re)vaccination of older people against vaccine preventable diseases (VPDs) may be an important preventive strategy for reducing the disease burden and health care costs in the aging population. Vaccines against influenza, pneumococcal disease, herpes zoster and pertussis are available, but only influenza is routinely offered to people aged 60 years and older in the Netherlands [8]. The diseases occur frequently among older people in the Netherlands [9] and vaccination against pneumococcal disease and herpes zoster is amongst others routinely offered to people aged 65 and 70 years of age respectively in the UK, while vaccination against pertussis is amongst others offered to people aged 65 in Belgium [10]. These vaccines are currently also being evaluated by the Dutch Health Council on their suitability to be routinely offered to older people in the near future.

As is the case in many European countries, general practitioners (GPs) carry out the national influenza vaccination program by selecting and inviting eligible patients from their registries and administering vaccination in the Netherlands. Moreover, previous research showed that the advice of the GP to get vaccinated was mentioned as the most important external influence on acceptance of vaccination by older people [11]. For this reason, they are the most likely choice in the Netherlands to administer vaccination when implementing new vaccination strategies for older people and to offer vaccination against infectious diseases other than influenza vaccination to elderly populations. Consequently, the feasibility of expanding the current program also depends on the willingness of GPs to organize and endorse new vaccination strategies. However, little is known about Dutch GPs’ attitude towards expanding the program for elderly people with additional vaccines, whether they would be willing to offer and administer those vaccines and under which circumstances.

Recently, a qualitative study was conducted in the Netherlands [12] exploring the attitude of GPs towards vaccinating older people and towards adding pneumococcal, herpes zoster and pertussis vaccination to the current program. In an effort to quantify the findings of this qualitative study, we conducted a cross-sectional study investigating the relative and combined strength of the identified factors and underlying beliefs in explaining the intention of GPs to vaccinate people aged 60 years and older against more infectious diseases than influenza.

## Methods

### Participants and procedure

In January 2015, all Dutch GPs (12.194 registered) working in 5068 general practices were invited to fill in an online questionnaire about the factors that influence their willingness to vaccinate people aged 60 years and older against infectious diseases other than influenza. GPs were asked to participate via a link in an e-mail sent by the National Influenza Prevention Program Foundation (SNPG), which coordinates the logistics of the influenza vaccination program (i.e. delivering vaccines to practices). Of the invited GPs 723 (6.0%) participated in this study. This type of study does not require ethics approval in the Netherlands because it does not fall under the Medical Research Involving Human Subjects act [13].

### The questionnaire

The questionnaire used for this study was based on an interview study among ten Dutch GPs, which explored GPs’ attitude towards vaccination of older people in general and potential future candidates for inclusion in the current influenza vaccination program for people aged 60 years and older [12]. The questionnaire consisted of 37 questions/statements about social cognitive factors, underlying beliefs, practical considerations, demographics, and characteristics of the GPs’ patient population. Variables were measured on 7-point Likert scales ranging from 1 = *totally disagree* to 7 = *totally agree*, unless otherwise indicated. Items measuring the same underlying construct were averaged into one single construct when internal consistency was sufficient (Cronbach’s alpha *α* > 0.60 or Pearson correlation coefficient *r* > 0.50). Items were recoded so that all effects pointed to the same direction. See Table 1 for an overview of the constructs and their internal consistency.

**Table 1:** Overview of constructs measured by the online survey

Variable	Number of items	Reliability	Example question
Intention	3	α = .88	I would be willing to vaccinate people aged 60 years and older against infectious diseases other than influenza.
Attitude	2	*r* = .64	I think vaccination as a preventive tool is: 1 = not very useful; 7 = very useful.
Attitude additional vaccination	1	n.a.	Offering additional vaccination other than influenza vaccination to people aged 60 years and older is necessary.
Attitude vaccination 80+	1	n.a.	Offering people 80 years and older some form of vaccination is still useful.
Attitude outbreak	1	n.a.	Vaccinating people 60 years and older with or without co-morbidities during an outbreak of an infectious disease is always useful.
Perceived severity (per infectious disease)	4	n.a.	How serious do you think are the different diseases for people aged 60 years and older? Influenza/ herpes zoster/ pneumonia/ pertussis: 1 = not severe at all; 7 = very severe. These are four separate items.
Selection comorbidity over age	3	α = .81	Vaccinating people on the basis of co-morbidities is favored over vaccinating people on the basis of age, irrespective of the infectious disease.
Prevention	1	n.a.	In general, prevention of illness has the preference over cure.
Prevention mortality	1	n.a.	Additional vaccines should primarily be focused on the prevention of death.
Prevention morbidity	1	n.a.	Additional vaccines should primarily be focused on the prevention of illness
Usefulness health benefits	1	n.a.	In the consideration to offer vaccination, individual health benefits are more important than cost-effectiveness on the population level.
Intention (per infectious disease)	3	n.a.	I would recommend healthy people in their 60s to get vaccinated against pneumococcal disease/ herpes zoster/ pertussis. These are three separate items.
Intention comorbidity (per infectious disease)	3	n.a.	I would recommend people in their 60s who have a comorbidity to get vaccinated against pneumococcal disease/ herpes zoster/ pertussis. These are three separate items.

n.a., not applicable

In addition to questions about their intention to vaccinate patients in their 60s against other infectious diseases than influenza, GPs were asked about their intention to recommend vaccination against pneumococcal disease, herpes zoster and pertussis to healthy people in their 60s and their intention to recommend vaccination of these diseases to people in their 60s with co-morbidity with separate items (see Table 1).

Moreover, GPs were asked to rank six arguments for offering vaccination to older patients. These arguments were identified as main arguments in the qualitative study preceding this cross-sectional study [12]. The arguments concerned health benefits for the individual, the severity of an infectious disease, vaccine effectiveness, side-effects of a vaccine, the outbreak of an infectious disease, and cost-effectiveness of vaccination. Furthermore, GPs were asked which of the proposed vaccines they considered to have the highest probability of being included in a program (pneumococcal/herpes zoster/pertussis vaccination/none of the above).

Next to that, practical considerations about offering additional vaccination were measured by asking about GPs’ suitability to apply additional vaccination, the importance of reimbursement for the implementation of a vaccination program, and preference for offering and administering additional vaccination at the same time as influenza vaccination, in the same national program, or outside of a national program.

Demographic variables were age, sex, and kind of practice (own practice, shared practice, integrated in health care setting, academic setting). Characteristics of GPs’ patient population were number of patients registered in the practice, an estimate of the proportion of patients aged 60 years and older registered in the practice, and the perceived incidence of influenza, pneumonia, herpes zoster, and pertussis among patients aged 60 years and older in their practice (1 = *never*; 7 = *very often*). See Additional file 1 for the entire questionnaire.

### Data analysis

Following a descriptive analysis of the sample (frequencies), univariate associations between intention and social cognitive factors, beliefs and practical considerations were tested with Spearman’s correlation test. To control for the false discovery rate (FDR) in multiple testing, the Benjamini-Hochberg [14] procedure was applied. Furthermore, paired z-tests were used to compare the intention to recommend vaccination against pneumococcal disease, herpes zoster and pertussis to people in their 60s when they are healthy and when they have co-morbidities.

In order to determine which factors contributed most to the prediction of GPs intention to offer additional vaccination and their attitude towards offering additional vaccination other than influenza vaccination to older people, prediction analyses were performed using randomForest [15]. RandomForest is an algorithm that predicts an outcome (intention, attitude) of an individual by means of several *predictor variables* (in our case social cognitive factors, beliefs, practical considerations, demographics and patient population characteristics). The relative importance of the predictor variables is assessed by determining how much the prediction error increases as a result of random permutation of the data for that variable. If a variable does not contribute to the prediction of the outcome, error estimates from the randomly permuted dataset will be about the same as for the original dataset, while prediction errors increase by permuting the data on variables that are crucial for the prediction of an outcome.

## Results

### Response and descriptive statistics

See Table 2 for demographics and patient population characteristics. Of the total sample, 463 (63.3%) were male, while 55% of the registered GPs were male. Participants had a mean age of 51.2 years (range 30 to 70), which was comparable to the mean age of the overall Dutch GP population (48.9 years). Comparisons with the total population of registered GPs are based on records from the general practitioners' registration poll of 2014 [16]. Most GPs reported to be part of a shared practice with several GPs (43.5%) or were working in their own practice (34.8%), instead of in a practice integrated into another health care setting (21.2%) or an academic setting (0.3%). On average, the number of patients registered in the practices was 3136 (range 220 to 17,000), with an estimated average of 750 patients above the age of 60 (range 0–4000). The average perceived incidence of the four diseases on a seven point Likert scale was 4.7 for pneumonia, 4.6 for influenza, 4.2 for herpes zoster, and 2.4 for pertussis. GPs ranked the severity of the infectious disease most often as the most important factor for recommending vaccination to people in their 60s (37.6% ranked it as most important), followed by the effectiveness of the vaccine (26.5%) and the expected health benefits for the individual (20.8%). Cost-effectiveness was the least often reported factor for recommending vaccination (43.2% ranked it as least important), followed by the side-effects of the vaccine (17.9%) and during an outbreak of the infectious disease (17.8%). Regarding the question of which vaccine candidate had the highest chance of being included in a vaccination program, 74.6% GPs chose pneumococcal vaccine, 10.5% herpes zoster vaccine, 4.2% pertussis vaccine, and 10.7% indicated that they considered that none of those vaccine candidates was likely to be included. The attitude towards vaccination as a preventive tool in general was significantly more positive than the attitude towards offering additional vaccination (M = 5.43 vs. M = 4.22, t(731) = 21.425, *p* < .001).

**Table 2:** Demographics and patient population characteristics

	Total GP sample (*N* = 723)	Total GP population (*N* = 12.194) in 2014
Gender		
Male	463 (63.3%)	55%
Female	269 (36.7%)	45%
Age (mean, SD)	51.2 (8.7)	48.9
Practice		
Own practice	34.8%	28%
Shared practice	43.5%	33%
Integrated in health care setting	21.2%	n.a.
Academic setting	0.3%	n.a.
Other	0.1%	39%
Work experience (in years, mean, SD)	20.2 (8.6)	n.a.
Total number of patients (mean, SD)	3136 (1631)	n.a.
Number of patients 60+ (mean, SD)	750 (500)	n.a.
Proportion of patients 60+ (mean, SD)	.25 (.14)	n.a
Perceived prevalence (1 = never; 7 = very often, mean)		
Influenza	4.6	n.a.
Herpes zoster	4.2	n.a.
Pneumonia	4.7	n.a.
Pertussis	2.4	n.a.

n.a., data not available

### Correlations between intention and attitude with items of the questionnaire

Here we report univariate associations of intention and attitude towards offering additional vaccination with the social cognitive factors, beliefs and practical considerations. In order to avoid false positive results we control the FDR at 5% (Table 3). In total, 134 associations among the 190 associations studied are declared significant at an FDR ≤ 5%, which corresponds to about seven or fewer false discoveries among the 134 associations (0.05*134 = 6.7). Both intention and attitude to offer additional vaccination showed significant univariate associations with almost all social cognitive factors and practical considerations considered. All associations were positive, except for the associations with selection based on co-morbidity instead of age, the belief that adding additional vaccination will complicate the organization of vaccination, and the belief that additional vaccination should primarily be based on the prevention of death. See Additional file 2 for all 190 associations.

**Table 3:** Selected pairwise associations for intention and attitude extra vaccination

Outcome variable (mean, SD)	Predictor variable	Mean	SD	Spearman correlation
Intention (4.68, 1.34)	Attitude extra vaccination	4.22	1.66	0.66
	Attitude	5.43	1.16	0.56
	Attitude vaccination outbreak	4.01	1.58	0.51
	Fit GP	5.57	1.46	0.48
	Perceived severity pneumonia	5.76	.85	0.46
	Attitude vaccination 80	4.88	1.52	0.45
	Perceived severity herpes zoster	4.54	1.29	0.41
	Prevention morbidity	5.31	1.37	0.40
	Selection comorbidity over age	5.10	1.23	−0.37
	Perceived severity flu	4.77	1.22	0.36
	Perceived severity pertussis	4.55	1.25	0.35
	Prevention	6.02	.99	0.31
	Difficult organisation	3.66	1.85	−0.25
	Outside program	4.88	1.47	0.20
	Reimbursement	6.51	.68	0.16
	One program	5.53	1.32	0.15
	Prevention mortality	4.31	1.78	−0.10
Attitude extra vaccination (4.22, 1.66)	Intention	4.68	1.34	0.66
	Attitude	5.43	1.16	0.46
	Perceived severity pneumonia	5.76	.85	0.42
	Attitude vaccination outbreak	4.01	1.58	0.40
	Attitude vaccination.80	4.88	1.52	0.40
	Selection comorbidity over age	5.10	1.23	−0.40
	Perceived severity herpes zoster	4.54	1.29	0.39
	Prevention morbidity	5.31	1.37	0.33
	Fit GP	5.57	1.46	0.32
	Perceived severity pertussis	4.55	1.25	0.31
	Perceived severity flu	4.77	1.22	0.30
	Prevention	6.02	.99	0.29
	Outside program	4.88	1.47	0.20
	Difficult organisation	3.66	1.85	−0.19
	Prevention mortality	4.31	1.78	−0.16
	Reimbursement	6.51	.68	0.12
	One program	5.53	1.32	0.12

False discovery rate (FDR) ≤ 5%, all correlations have *p* ≤ .001

*SD* standard deviation

### Effect of co-morbidity on intention to vaccinate

There were significant differences between the scores for intention to recommend vaccination for healthy people aged 60 (pneumonia M = 4.1, SD = 1.7; herpes zoster M = 3.3, SD = 1.7; pertussis M = 3.3, SD = 1.5) and for people aged 60 with co-morbidities (pneumonia M = 5.3, SD = 1.4; herpes zoster M = 4.4, SD = 1.6; pertussis M = 4.3, SD = 1.6): the *p*-values of the z-tests are all well below 0.001 for pneumonia, herpes zoster and pertussis. The results suggest that the presence of co-comorbidities increases the intention of GPs to recommend vaccination against the three infectious diseases to people in their 60s. Intention with or without co-morbidities was highest for pneumonia, followed by herpes zoster and pertussis.

### Prediction analysis

**Fig. 1:**
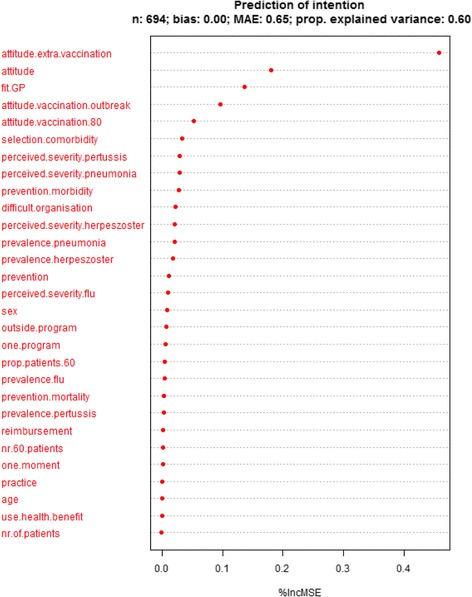
Prediction of intention to offer additional vaccination to people aged 60 years and older other than influenza vaccination. MAE: mean absolute error. %IncMSE percentage increase in mean absolute error

**Fig. 2:**
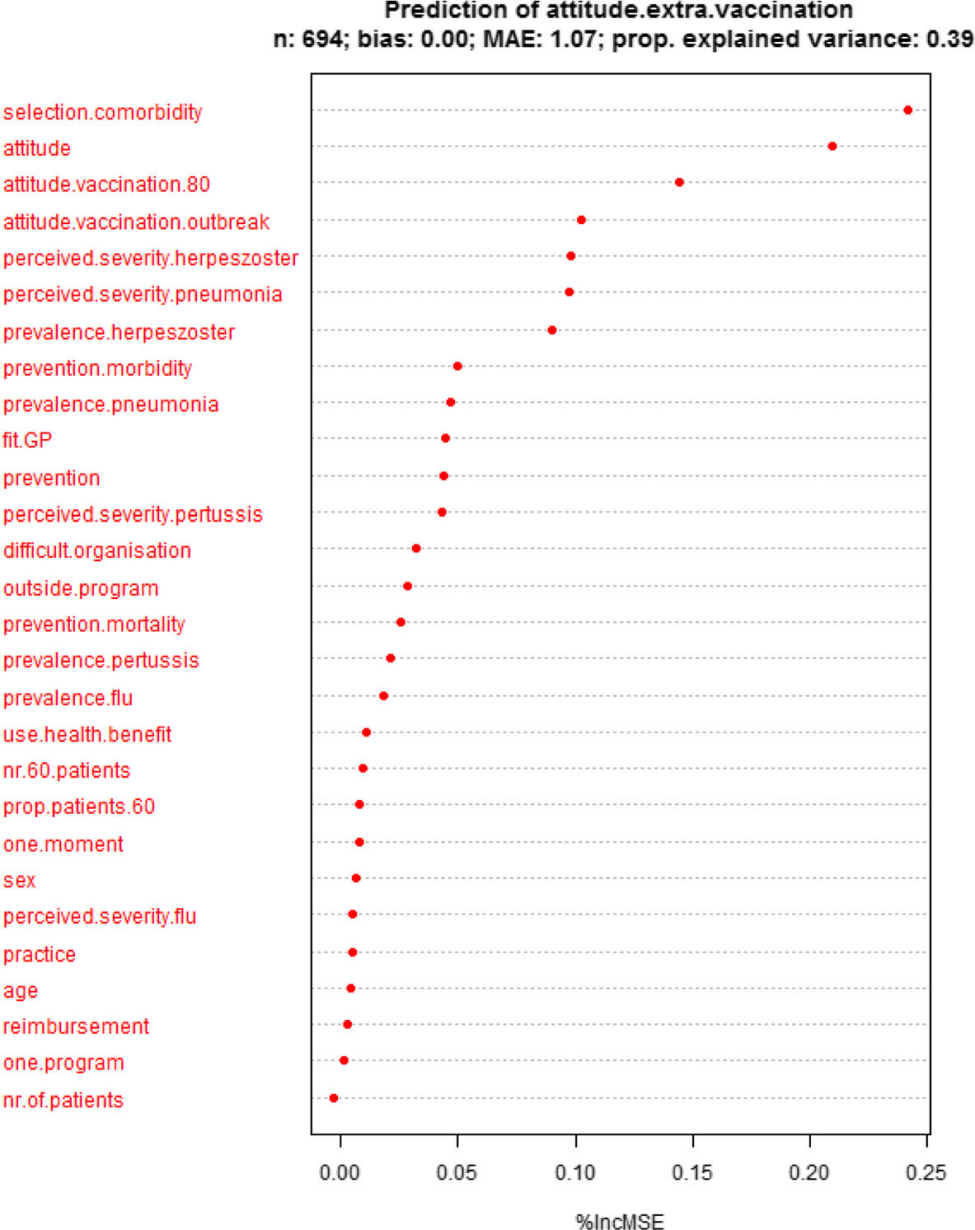
Prediction of attitude towards offering additional vaccination to people aged 60 years and older other than influenza vaccination. MAE: mean absolute error. %IncMSE percentage increase in mean absolute error

Due to missing values, the randomForest algorithm was performed for 694 participants (see Figs. 1 and 2). The variables included in the prediction analysis with intention to offer additional vaccination other than influenza as outcome explained 60% of the variance in intention. Attitude towards offering additional vaccination other than influenza vaccination to older people, the general attitude towards vaccination as a preventive tool, the practical consideration of GPs being suitable to administer additional vaccination, and the attitude towards offering older people vaccination during an outbreak of the infectious disease are among those with the largest predictive value for the intention to vaccinate people aged 60 years and older against infectious diseases other than influenza. The variables included in the prediction analysis with attitude towards offering additional vaccination as an outcome explained 39% of the variance in attitude. Factors with the largest predictive value for attitude include the selection of vaccination for older individuals on the basis of co-morbidities instead of age, general attitude towards vaccination as a preventive tool, the attitude towards offering people aged 80 years and older vaccination, the attitude towards offering older people vaccination during an outbreak of the infectious disease, and the perceived severity of herpes zoster and pneumonia, as well as the prevalence of herpes zoster.

## Discussion

This study found that the intention of GPs to offer additional vaccination to people aged 60 years and older other than influenza vaccination was mainly predicted by their attitude towards offering additional vaccination, their attitude towards vaccination as a preventive tool in general, and their attitude towards offering vaccination during the outbreak of an infectious disease. On average, GPs seem to be positive about vaccination as a preventive tool in general, but a bit less positive about offering additional vaccination to people aged 60 years and older. Our findings further suggest that GPs are more willing to recommend vaccination against pneumococcal disease, herpes zoster and pertussis to patients in their 60s when those patients have co-morbidities, with the most positive intention for pneumococcal disease. The attitude of GPs to offer additional vaccination was amongst others predicted by the perceived severity of pneumococcal disease and the perceived severity and prevalence of herpes zoster. Research from the US, Australia and Italy has also shown that GPs had a positive attitude towards recommending influenza and pneumococcal vaccination to older patients and that these diseases were perceived to be serious for older people [17–20]. Moreover, research from the United States has shown that GPs recommend herpes zoster vaccination less often to their older patients than they do influenza and pneumococcal vaccination [21], despite the recognition of GPs that herpes zoster can cause prolonged suffering among older individuals [22]. This is in accordance with our finding that most GPs indicate the pneumococcal vaccine as the one among the three vaccine candidates with the highest chance to be added to the national program, and that only few GPs consider herpes zoster vaccination and pertussis vaccination for inclusion. This is also in line with the estimated disease burden of the respective diseases [23], as well as with the prioritization of Dutch older adults, who had indicated the highest acceptance for pneumococcal vaccination and the lowest for pertussis vaccination [11]. Consequently, the intention of Dutch GPs to offer additional vaccination to older people will probably depend on their perceived severity of the diseases and their disease burden. GPs ranked the severity of an infectious disease most frequently as an important argument for offering vaccination to older people, followed by the effectiveness of the vaccine, and the expected health benefits for the individual. Cost-effectiveness was ranked as the least important argument for offering vaccination. Side effects of the vaccine and the outbreak of an infectious disease were also ranked lower in the importance for the decision to offer vaccination. Previous research from the US had shown that beliefs about vaccine effectiveness, the risk for illness, and cost-effectiveness of a vaccine were strongly associated with the recommendation of pneumococcal vaccination by GPs [17].

The selection of vaccination for older individuals based on co-morbidities instead of age, also appeared as one of the main predictors of the attitude of GPs to offer additional vaccination to older people. Although it was difficult to interpret its influence since it shows a negative univariate association with attitude to offer additional vaccination, we think that selection on comorbidity is preferred. The contradiction was very likely due to the way the statement was framed: “Vaccinating people on the basis of co-morbidities is favored over vaccinating people on the basis of age, irrespective of the infectious disease” contains a weighing up of two alternatives. In order to better understand this contradiction, we presented this item as two separate statements to a group of GPs (*N* = 99). Results showed that selection of vaccination for older individuals on the basis of co-morbidity was favored over selection on the basis of age. Attitude to offer additional vaccination to people aged 60 years and older other than influenza was further predicted by their general attitude towards vaccination as a preventive tool, as well as by their attitude towards offering vaccination to patients in their 80s and during the outbreak of a disease.

Moreover, intention was predicted by whether GPs think that they are the suitable professionals to offer additional vaccination to older people. Considering the importance of the role of GPs in expanding the current vaccination program for older people, it is a positive sign that they consider themselves suitable for offering and applying additional vaccination. In the qualitative study preceding this study, GPs explained that they were suitable to implement new vaccination strategies, because of their successful implementation of the influenza vaccination program, their knowledge on the disease histories of their patients, and their role as health educators that are trusted by older patients [12].

Influenza vaccination uptake rates among older people are suboptimal in Europe [24]. Previously reported barriers to administering vaccination to adults have been a lack of physicians’ knowledge about vaccination [25], poor vaccine supply [26], cost of vaccinations [27], and practice barriers, such as competing priorities in care especially in the presence of acute or chronic problems [28, 29]. The most common barriers for vaccination uptake among patients were a lack of knowledge about the benefits of vaccination [30], concerns about vaccine safety [31, 32] and when vaccination is not recommended by physicians [11, 32, 33]. In the light of our findings, it seems to be especially important to inform GPs about available vaccines for older people and to provide them with evidence-based information on the incidence and severity of the targeted infectious diseases among older people. The finding that GPs have a more positive intention to offer pneumococcal, herpes zoster and pertussis vaccination to older patients, when they have a co-morbidity is in line with research showing that co- and multi-morbidity lead to higher susceptibility towards infectious diseases in older people [4]. However, older age on its own also increases the incidence of serious complications and mortality associated with infection [4, 34, 35]. Identifying the influence of co-morbidities on the disease burden of infectious diseases poses methodological challenges [23] since data on the occurrence of comorbidity is often missing. Policy advisers should therefore clearly state evidence-based implications, explaining why recommendation of vaccination is based on age rather than on co-morbidity. For example, research has suggested that vaccination against herpes zoster should be focused on specific age groups, since the disease burden was relatively low for people aged 50 years and older, but increased considerably for people aged 75 years and older [23].

A limitation that should be mentioned is that the survey length was kept to a minimum and the variables were for the most part measured by one single item, which might have lowered measurement specificity. This was done to achieve a higher response rate. Still, response bias is likely with the low response rate of 6% and we only have limited data to compare participating GPs to the total population of Dutch GPs. Moreover, generalization of the findings to other countries should be treated with caution since vaccination policies, as well as vaccination acceptance differ among countries [36].

## Conclusions

In conclusion, GPs seem to be positive about offering at least some of the additional vaccinations to older people, when infectious diseases are perceived as severe and prevalent. They also feel suitable to administer additional vaccination. In order to ensure a positive attitude of GPs towards informing about and administering additional vaccinations to older people, they need to have clear guidelines, including evidence-based information about severity and incidence of the diseases, the effectiveness and health benefits of the vaccines, as well as about advising vaccination based on high-risk groups.

## Additional files

Additional file 1: Questionnaire. (DOCX 28 kb)

Additional file 2: All correlations between intention and attitude with items of the questionnaire. (XLSX 24 kb)

## Abbreviations

FDR: False discovery rate
GP: General practitioner
SNPG: National Influenza Prevention Program Foundation
VPD: Vaccine preventable disease
