# Supplementary material for: The intention of Dutch general practitioners to offer vaccination against pneumococcal disease, herpes zoster and pertussis to people aged 60 years and older
Source: BMC Geriatr. 2017 Jun 7;17:122. doi: 10.1186/s12877-017-0511-7 (PMC5463354; doi:10.1186/s12877-017-0511-7)
Supplement: Supplementary file 1 — Questionnaire. (DOCX 28 kb) [file 12877_2017_511_MOESM1_ESM.docx]

**Questionnaire**

**The intention of Dutch general practitioners to offer vaccination against pneumococcal disease, herpes zoster and pertussis to people aged 60 years and older**

**Attitude towards prevention and (influenza) vaccination among people aged 60 years and older**

Please indicate which response reflects your opinion best about the following statements.

1. I think vaccination as a preventive tool is:

- 1 = Not very useful – 7 = Very useful

2. I think the current vaccination program is:

- 1 = Not very useful – 7 = Very useful

3. In general, prevention of illness has the preference over cure.

- 1 = Totally disagree – 7 = Totally agree

4. Offering additional vaccination other than influenza vaccination to people aged 60 years and older is necessary.

- 1 = Totally disagree – 7 = Totally agree

5. Additional vaccines should primarily be focused on the prevention of death.

- 1 = Totally disagree – 7 = Totally agree

6. Additional vaccines should primarily be focused on the prevention of illness.

- 1 = Totally disagree – 7 = Totally agree

7. Vaccinating people on the basis of co-morbidities is favored over vaccinating people on the basis of age, irrespective of the infectious disease.

- 1 = Totally disagree – 7 = Totally agree

8. Vaccinating people on the basis of age is favored over vaccinating people on the basis of co-morbidities, irrespective of the infectious disease.

- 1 = Totally disagree – 7 = Totally agree

9. Vaccinating people on the basis of co-morbidities is favored over vaccinating the whole population of people aged 60 years and older, irrespective of the infectious disease.

- 1 = Totally disagree – 7 = Totally agree

10. In the consideration to offer vaccination, individual health benefits are more important than cost-effectiveness on the population level.

- 1 = Totally disagree – 7 = Totally agree

11. Offering people 80 years and older some form of vaccination is still useful.

- 1 = Totally disagree – 7 = Totally agree

12. Vaccinating people 60 years and older with or without co-morbidities during an outbreak of an infectious disease is always useful.

- 1 = Totally disagree – 7 = Totally agree

13. Can you indicate below how severe you think the different diseases are for people 60 years and older?

|  | Not severe at all | Not severe | Rarely severe | Neutral | A bit severe | Severe | Very Severe |
| --- | --- | --- | --- | --- | --- | --- | --- |
| Influenza |  |  |  |  |  |  |  |
| Herpes zoster |  |  |  |  |  |  |  |
| Pneumococcal disease |  |  |  |  |  |  |  |
| Pertussis |  |  |  |  |  |  |  |

14. Please order the below mentioned arguments in the order of importance for recommending vaccination to people aged 60 years and older. 1 is the most important reason and 6 is the least important reason.

|  | Rank |
| --- | --- |
| a. Expected health benefits for the individual |  |
| b. Severity of the disease |  |
| c. Vaccine effectiveness |  |
| d. Side-effects of the vaccine |  |
| e. At an outbreak of a disease |  |
| f. Cost-effectiveness of a vaccine |  |

**Intention to vaccinate**

1a. I would be willing to vaccinate people aged 60 years and older against infectious diseases other than influenza.

- 1 = Totally disagree – 7 – Totally agree

1b. If I am asked to vaccinate people aged 60 years and older against infectious diseases other than influenza, I will do it.

- 1 = Totally disagree – 7 – Totally agree

1c . I am planning to vaccinate people aged 60 years and older against infectious diseases other than influenza.

- 1 = Totally disagree – 7 – Totally agree

2a. I would recommend healthy people in their 60s to get vaccinated against herpes zoster.

- 1 = Totally disagree – 7 – Totally agree

2b. I would recommend people in their 60s who have a comorbidity to get vaccinated against herpes zoster.

- 1 = Totally disagree – 7 – Totally agree

3a. I would recommend healthy people in their 60s to get vaccinated against pertussis.

- 1 = Totally disagree – 7 – Totally agree

3b. I would recommend people in their 60s who have a comorbidity to get vaccinated against pertussis.

- 1 = Totally disagree – 7 – Totally agree

4a. I would recommend healthy people in their 60s to get vaccinated against pneumococcal disease.

- 1 = Totally disagree – 7 – Totally agree

4b. I would recommend people in their 60s who have a comorbidity to get vaccinated against pneumococcal disease.

- 1 = Totally disagree – 7 – Totally agree

**Practical considerations**

1. The general practitioner is the most suitable person to also offer possible additional vaccinations other than influenza vaccination to people aged 60 years and older.

- 1 = Totally disagree – 7 = Totally agree

2. A reimbursement for implementing a vaccination program would be essential.

- 1 = Totally disagree – 7 = Totally agree

3. Offering additional vaccination would complicate the organization in practice.

- 1 = Totally disagree – 7 = Totally agree

4. Possible new vaccines should be administered at the same moment as the influenza vaccine.

- 1 = Totally disagree – 7 = Totally agree

5. It would be desirable to combine new and existing vaccines in one (national) program.

- 1 = Totally disagree – 7 = Totally agree

6. It would be difficult to offer additional vaccines outside of a national vaccination program.

- 1 = Totally disagree – 7 = Totally agree

7. Which of the following vaccines has the highest probability to be included in a program according to you?

- Herpes zoster vaccine
- Vaccine against pneumococcal disease
- Pertussis vaccine
- None of the above mentioned vaccines

8. Are there other vaccines than vaccination against influenza, herpes zoster, pertussis and pneumococcal disease that you would want to offer to people 60 years and older? (open question)

______________________

Now we would like to as you a number of general questions about you and the practice where you work.

**General questions**

- What is your age?
- What is your sex?
- What is your position in the practice where you work?
  - General practitioner
  - Assistant
  - Other
- In case you are a general practitioner, how long have you been working as general practitioner?
- How many patients are registered in your practice?
- How many patients are aged 60 years or older? (An estimation is sufficient)
- In what kind of practice are you working?
- Own practice
- Shared practice
- Integrated in health care setting
- Academic setting
- Other

Could you indicate how often you see the disease influenza, herpes zoster, pertussis and pneumococcal disease per year among people aged 60 years and older in your practice?

| I see: | Never | Barely | Occasionally | Sometimes | Regularly | Often | Very often |
| --- | --- | --- | --- | --- | --- | --- | --- |
| Influenza among people 60+ |  |  |  |  |  |  |  |
| Pertussis among people 60+ |  |  |  |  |  |  |  |
| Pneumonia among people 60+ |  |  |  |  |  |  |  |
| Herpes zoster among people 60+ |  |  |  |  |  |  |  |
